# Spectrophotometric assays for total antioxidant capacity (TAC) in dog serum: an update

**DOI:** 10.1186/s12917-016-0792-7

**Published:** 2016-08-15

**Authors:** Camila Peres Rubio, Josefa Hernández-Ruiz, Silvia Martinez-Subiela, Asta Tvarijonaviciute, José Joaquin Ceron

**Affiliations:** 1 Interdisciplinary Laboratory of Clinical Analysis Interlab-UMU, Faculty of Veterinary Medicine, Regional Campus of International Excellence ‘Campus Mare Nostrum’, University of Murcia, 30100 Espinardo, Murcia Spain; 2 Department of Plant Biology (Plant Physiology), Faculty of Biology, University of Murcia, Murcia, Spain; 3 Department of Animal Medicine and Surgery, Veterinary School, University Autonoma of Barcelona, Barcelona, Spain

**Keywords:** Antioxidant activity, Canine serum, Human serum, Cupric, Ferric, Oxidative stress, Trolox

## Abstract

The aim of this review is to study the main spectrophotometric methods used to evaluate total antioxidant capacity (TAC) in serum samples of dogs. Total antioxidant capacity (TAC) is an analyte frequently used to assess the antioxidant status of biological samples and can evaluate the antioxidant response against the free radicals produced in a given disease. Trolox equivalent antioxidant capacity (TEAC), ferric reducing ability of plasma (FRAP), and cupric reducing antioxidant capacity (CUPRAC) are different assays described to determine TAC of a sample. This review explains the basis of each assay and their application in the determination of TAC in dogs, and also provides selected information about reports in humans for comparative purposes. It is concluded that, ideally, various different assays integrated in a panel should be used for TAC evaluation, since depending on the assay performed TAC results can be markedly different.

## Background

An antioxidant is defined as “any substance that, when presented at low concentration compared to those of an oxidizable substrate (proteins, lipids, carbohydrates, and DNA), significantly delays, or prevents oxidation of that substrate” [1, 2]. The main function of antioxidants is to protect the body against the destructive effects of free radicals damage [3].

Free radicals may be generated in cells and tissues from internal (such as inflammation, diseases or metabolism) or external sources (irradiation, pollution, food, drugs), or as a consequence of decreased protective capacity [1]. In any case, an increase in free radicals production can originate oxidative damage [4, 5].

## General concepts

Total antioxidant capacity (TAC) is the measure of the amount of free radicals scavenged by a test solution [4], being used to evaluate the antioxidant capacity of biological samples [3, 6, 7].

### Classification of different TAC assays

Assays to measure TAC can be direct, which are based on the ability to inhibit the oxidation of a substance. One of the most commonly used direct assays is the Trolox (6-hydroxy-2,5,7,8-tetramethylchroman-2-carboxylic acid) equivalent antioxidant capacity (TEAC) with modifications mainly based on period of time used for measurement, and radical formed [8–10]. The oxygen radical absorbance capacity (ORAC) assay is another direct assay described [11]. On the other hand, there are indirect assays, such as the ferric reducing ability of plasma (FRAP) [12], and the cupric reducing antioxidant capacity (CUPRAC) [13], which are based on determination of the ability of a sample to reduce a metal complex [6]. Trolox equivalent antioxidant capacity (TEAC), FRAP and CUPRAC are spectrophotometric, whereas ORAC is a fluorometric assay.

On the basis of the chemical reactions involved, TAC assays can be also divided into two categories: hydrogen atom transfer (HAT) based methods or on single electron transfer (SET) based methods. The HAT-based methods measure the ability of an antioxidant to quench free radicals by hydrogen donation. These methods are pH independent and are usually quite rapid, typically completed in seconds to minutes. One example is the ORAC assay. The SET-based methods detect the ability of a potential antioxidant to transfer one electron to reduce any compound, including metals, carbonyls, and radicals. They are pH dependent and based on percent decrease in product rather than kinetics, being usually slower than the HAT-based methods [6]. Ferric reducing ability of plasma (FRAP), CUPRAC and TEAC assays are based on SET reaction mechanisms [14, 15].

### Main advantages and limitations of TAC assays

One major advantage of TAC assays is that, by definition, estimate the antioxidant components of a sample in a global way. Measuring each antioxidant component individually is labor-intensive and time-consuming, requiring complex and costly techniques [9]. Other advantages of using TAC assays include simplicity of the techniques, low cost per sample, speed of reactions and possibility to be performed using automated, semi-automated, or manual methods [3, 6].

However, measurement of only TAC can provide limited information about the antioxidant status, because TAC assays do not measure all antioxidant components. For example, they do not evaluate the role of important enzymes such as superoxide dismutase, glutathione peroxidase, and catalases [6, 16, 17]. Therefore, plasma antioxidant capacity provides a reductionist modelling of an in vivo situation, and, therefore caution is needed in the interpretation of results [18].

### What is the best assay for TAC measurements?

Despite spectrophotometric measurements of TAC are easy and fast, the answer about what would the ideal assay for TAC measurement is controversial due to inherent limitations of each method and because each method measures different TAC components [19]. This situation could explain the variability in results found with different TAC assays. Cao and Prior [20] found no correlation between ORAC and TEAC or between FRAP and TEAC in serum of healthy humans. This agrees with other reports indicating that different results can be obtained when different assays to measure TAC are applied to human serum or plasma [21, 22]. When dogs with cardiac disorders (mitral endocardiosis and dilated cardiomyopathy) where compared with healthy dogs, no significant differences were found in serum TEAC, but lower values were found in diseased dogs when FRAP was used [23]. In accordance with Huang et al. [24], the major problem is the lack of a method that can measure in an accurate way the global antioxidant capacity of biological samples.

### Current situation and objective of the review

There are a number of reviews regarding the measurement of TAC in foods and biological fluids from humans presenting detailed description of the assay procedures, reaction kinetics, and also advantages and limitations of the different methods [6, 24–29]. This paper studies the spectrophotometric assays which currently are most frequently used to measure TAC in serum samples (TEAC, FRAP and CUPRAC) with a special interest in dogs, and also provides selected information about reports in human medicine for comparative purposes.

It is expected that the information provided here can contribute to increase the use of TAC measurements, especially in companion animal’s research and therefore more information could be provided in the future about the behavior of different assays in selected diseases and their clinical use.

## TEAC assay

Trolox equivalent antioxidant capacity (TEAC) assay is generally based on the ability of antioxidants presenting in a sample in reduce or inhibit oxidized products generated in the assay. It is based on the principle that when ABTS (2,2’-azino-bis(3-ethylbenz-thiazoline-6-sulfonic acid) is incubated with a proper chemical, an ABTS radical (ABTS^•+^) is formed. The ABTS^•+^ has a blue-green color, with maximum absorptions at 650, 734 and 820 nm. Antioxidants in the sample reduce ABTS^•+^ suppressing this color production to a degree that is proportional to their concentrations [8]. In human plasma, TEAC measures mainly albumin (that represents 43-53 % of the total value) and uric acid (representing 33 %). In addition, it measures ascorbic acid, α-tocopherol, and bilirubin [8, 9].

Reaction rate is commonly calibrated with Trolox, a water soluble analogue of vitamin E, and assay results are expressed in mmol Trolox equivalent/L [8]. This method is suitable for automation permitting rapid throughput of samples and various kits based on this method are commercially available.

### TEAC versions

There are different versions of TEAC assay, with variations in how the oxidized radical is generated and times of reaction used [30]. The method of Miller et al. [8] uses metmyoglobin, Arnao et al. [30–32] developed a method for foods and plant material using the horseradish peroxidase enzyme (HRP), Re et al. [10] used potassium persulfate, and Erel [9] used H_2_O_2_ in acid medium for producing the oxidized radical.

**Fig. 1:**
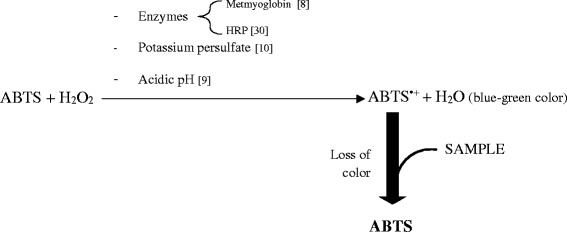
Mainly differences in ABTS^**•** +^ generation between the different TEAC assays

The Fig. 1 summarizes the principal variations of the TEAC method that have been described.

### Advantages and limitations

The TEAC presents some advantages [8, 9, 14]:

- operational simplicity
- the radical ABTS is soluble in both aqueous and organic solvent media enabling the simultaneous determination of hydrophilic and lipophilic antioxidants
- can be automated

On the other hand, it also presents limitations such as [15, 16]:

- the radical ABTS used in the method does not represent a physiological radical source
- may take a long time to reach an endpoint.

### Validation data

According to the reported validation data, presented in Table 1, TEAC assays described to determine the TAC in sample of human and dogs have good reproducibility.

**Table 1:** Coefficient of variation (CV) of the TEAC assay and variants

Method	Specie	Intra-assay CV (%)	Inter-assay CV (%)	Reference
ABTS, metmyoglobin and H_2_O_2_	Human	0.54–1.59	3.6–6.1	[8]
Kit - Miller et al. [8]	Human	2.5	5.0	[20]
ABTS and H_2_O_2_	Human	1.3–2.5	1.5–2.9	[9]
Kit - Erel [9]	Human	No reported	8.9	[21]
Kit - Miller et al. [8]	Dogs	2.8	No reported	[33]

TEAC, Trolox equivalent antioxidant capacity; ABTS, 2,2’- azino - bis(3 - ethylbenz - thiazoline - 6 - sulfonic acid); H_2_O_2_, hydrogen peroxide

### Studies in human

In studies made with commercial kits based on the method of Miller et al., Glantzounis et al. [34] and Zulfikaroglu et al. [35] observed significant decreased TAC values in human patients submitted to laparoscopic cholecystectomy. Patients with metabolic syndrome [36] and with prediabetes [37] showed TAC values significantly lower than healthy subjects. In addition, non-surviving septic patients had higher serum TAC levels than surviving ones [38].

Using the method developed by Erel, Koksal and Kurban [39] reported no significant changes in the serum TAC from human patients before and after abdominal wall hernias resolution. However, significant differences in the values between the surgical techniques used for the hernias resolution were observed. *Helicobacter pylori* infection produced a decrease in TAC values [40]. In other studies there was no difference in TAC values in patients suffering from brucellosis [41], but TAC was increased in patients with acute appendicitis [42].

### Studies in dogs

Results of studies that determined the TAC by the TEAC methods in serum samples of dogs are shown in Table 2. In this table, it can be observed that TAC determined with the method developed by Erel decreased after surgery, anesthesia, in visceral leishmaniosis, and after vaccination against canine monocytic ehrlichiosis; and increased in demodicosis and parvoviral enteritis. When the method described by Miller et al. was used, dogs with *Babesia vogeli*, atopic dermatitis and heart diseases showed no significant differences when compared with healthy ones.

**Table 2:** Studies determining the TAC using the various TEAC methods in serum samples of dogs

Disease/effect studied	Method	Comparison with healthy animals	Reference
Established range in healthy beagle	Kit - Miller et al. [8]	-	[33]
*Babesia vogeli*	Kit - Miller et al. [8]	No difference	[43]
Atopic dermatitis	Kit - Miller et al. [8]	No difference	[44]
Heart diseases	Miller et al. [8]	No difference	[23]
Parvoviral enteritis	Erel [9]	Increased	[45]
Demodicosis	Erel [9]	Increased	[46]
Visceral leishmaniosis	Erel [9]	Decreased	[47]
After surgery	Erel [9]	Decreased	[48]
After anaesthetized with isoflurane	Erel [9]	Decreased	[49]
Sarcoptic mange	Erel [9]	No difference	[50]
After vaccination against canine monocytic erlichiosis	Erel [9]	Decreased	[51]

*TAC* total antioxidant capacity; *TEAC*, Trolox equivalent antioxidant capacity

## FRAP assay

**Fig. 2:**
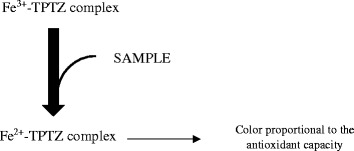
An overview of FRAP reaction

Ferric reducing ability of plasma (FRAP) assay is based on the principle of reduction of ferric-tripyridyltriazine (Fe^3+^-TPTZ) complex to ferrous tripyridyltriazine (Fe^2+^-TPTZ) by the antioxidants of a sample at low pH [12]. The end product (Fe^2+^-TPTZ) has blue color with absorption maximum at 593 nm and the change in absorbance is related to the antioxidant capacity of the plasma (Fig. 2).

Uric acid is the main component (can reach up to 60 %) of FRAP in human plasma. In addition, this assay measures ascorbic acid, bilirubin, and α-tocopherol [12].

For calibration, aqueous solutions of known Fe (FeSO_4._7H_2_O) concentration in the range of 100 to 1000 μmol/L are used, and the values are expressed as μmol/L Fe^2+^ [12]. This assay can be performed using automated, semi-automated, or manual methods [12, 52].

### Advantages and limitations

The FRAP assay has some advantages [12, 52]:

- no need of highly specialized equipment or skills, or strict control of timing and reaction conditions
- quick and simple to perform and can be easily automated
- reagents are inexpensive and sample pre-treatment is not required
- highly reproducible over a wide concentration range

However, FRAP reaction conditions are far from physiological to measure the TAC (i.e. to maintain iron solubility requires an acidic pH (3.6)) [26]. In addition, it does not measure the antioxidants containing thiol groups and only measures the reducing capability based upon the ferric ion, which is described by some authors not being relevant to antioxidant activity [7, 29].

### Validation data

Few data are published on the FRAP assay validation, being based on studies with human samples. Benzie and Strain [12] reported an intra- and inter-assay CV of <1.0 % and < 3.0 %, respectively; and a recovery of 91-112 %. Jansen and Ruskovska [21] indicated an inter-assay CV of 11.4 %.

### Studies in human

Jansen and Ruskovska [21] observed a significant correlation between the TAC values measured by FRAP assay and the TEAC assay developed by Miller et al. in healthy males. Jansen et al. [53] and Haldar et al. [54] observed no differences in the FRAP values when compared smokers with no-smokers, and omnivores with vegetarians, respectively. There was no difference in serum concentrations of FRAP in patients with oral carcinoma before and after the treatment [55]. Hyperglycemia leads to lower FRAP values when compared to healthy subjects [56] and elderly physically disabled patients had lower FRAP values when compared to healthy ones [57].

### Studies in dogs

There is no influence of age on FRAP as older dogs have similar FRAP values than the young ones [58].

Serum FRAP was significantly higher in dogs with heart disease than in control animals [23]. In addition, obese dogs submitted to a weight loss program had higher FRAP values when compared to the baseline [59]; however, the values were not different between healthy dogs and dogs submitted to a weight gain program [60].

Dogs with mammary carcinoma of low-grade malignancy had higher FRAP levels when compared with dogs with high-grade mammary carcinoma and healthy dogs [61]. However, no difference in the FRAP levels were observed between dogs with lymphomas and healthy dogs [62].

FRAP increased in plasma of dogs exposed to one therapeutic dose of asoxime chloride, an oxime reactivator used for counteracting intoxication by nerve agents [63] and after supplementation with antioxidant blend [64]. Dogs that received lidocaine or placebo during ovariohysterectomy had no different FRAP values between the treatments and the different times of sampling (before premedication, during suturing, after extubation and after surgery) [65].

## CUPRAC assay

**Fig. 3:**
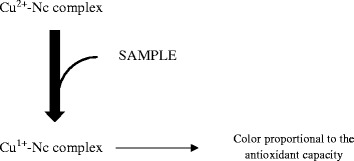
An overview of CUPRAC reaction

This assay evaluates the capacity of the antioxidants of a sample to reduce the Cu^2+^ to Cu^1+^ in the presence of a chelating agent as summarized in Fig. 3. These chelators form colored stable complexes with Cu^1+^ that have a maximum absorption at 450–490 nm [13].

The CUPRAC assay measures the thiol-group antioxidants and other plasma antioxidants such as ascorbic acid, α-tocopherol, β-carotene, uric acid, albumin, and bilirubin [13].

### CUPRAC versions

The CUPRAC assays differ mainly in the chelating agent used, that can be bathocuproine (BC), bathocuproinedisulfonic acid disodium salt (BCS), or neocuproine (Nc). An assay based on the Cu^2+^ reduction using BC is commercially available [66].

### Advantages and limitations

The method presents some advantages [13, 67]:

- color development is relatively fast
- reagents are relatively stable, cheap, and accessible
- suitable for automation, does not require great expertise or the use of expensive equipment
- the redox reaction producing colored species is carried out at pH 7 buffer (close to that of physiological pH)
- thiols are detected
- can simultaneously measure lipophilic and hydrophilic antioxidants.

On the other hand, the assay does not measure antioxidant enzymes and depending on the CUPRAC version, it can require longer times of measurement and previous sample preparation [13].

### Validation data

The intra- and inter-assay CVs of the CUPRAC method using Nc for human serum were 0.7 and 1.5 %, respectively [13], and using BCS were between 0.9 and 5.6 %, respectively [67, 68]. In serum of dogs, the intra- and inter-assay of the CUPRAC method using BCS were between 2 and 9 %, respectively [69].

### Studies in human

Cupric ion reducing antioxidant capacity (CUPRAC) was significantly correlated with the FRAP and TEAC in healthy subjects [67]. Gosmaro et al. [68] observed lower CUPRAC values in patients after hemo-dialysis treatment.

### Studies in dogs

Significant lower CUPRAC values were found in serum of dogs with inflammatory bowel disease when compared to healthy dogs [69].

## Conclusion

In this review, we have analyzed the main assays for TAC measurements with special emphasis on their advantages and disadvantages, and the studies where they have been applied in dogs and humans. Based on this analysis, it could be recommended that, if possible, until an ideal reference method is found, various different assays integrated in a panel should be used for TAC evaluation. This recommendation is supported by the fact that depending on the assay performed, the result of TAC could be markedly different. In addition, the combination of TAC assays with more specific analysis of individual antioxidants would provide a wider picture of the antioxidant status.

## Abbreviations

ABTS, 2,2’- azino - bis(3 - ethylbenz - thiazoline - 6 - sulfonic acid); ABTS^•+^, 2,2’- azino - bis(3 - ethylbenz - thiazoline - 6 - sulfonic acid) radical; BC, bathocuproine; BCS, bathocuproinedisulfonic acid disodium salt; CUPRAC, cupric reducing antioxidant capacity; CV, coefficient of variation; DNA: deoxyribonucleic acid; Fe^2+^-TPTZ, ferrous tripyridyltriazine; Fe^3+^-TPTZ, ferric-tripyridyltriazine; FRAP, ferric reducing ability of plasma; GSH, reduced glutathione; H_2_O_2_, hydrogen peroxide; HAT, hydrogen atom transfer; HRP, horseradish peroxidase enzyme; Nc, neocuproine; ORAC, oxygen radical absorbance capacity; SET, single electron transfer; TAC, total antioxidant capacity; TEAC, Trolox equivalent antioxidant capacity
